# Addition of an induction regimen of antiangiogenesis and antitumor immunity to standard chemotherapy improves survival in advanced malignancies

**DOI:** 10.1007/s12032-012-0301-1

**Published:** 2012-07-19

**Authors:** Eduardo Lasalvia-Prisco, Pablo Goldschmidt, Felipe Galmarini, Silvia Cucchi, Jesús Vázquez, Martha Aghazarian, Eduardo Lasalvia-Galante, Wilson Golomar, William Gordon

**Affiliations:** 1 Interdoctors Network, Montevideo, Uruguay; 2 (F) University of the Republic of Uruguay, Montevideo, Uruguay; 3 (F) National Cancer Institute, Montevideo, Uruguay; 4 National Cancer Institute, Montevideo, Uruguay; 5 Interdoctors Network, Paris, France; 6 Interdoctors Network, Buenos Aires, Argentina; 7 Interdoctors Network, North Miami Beach, FL USA

**Keywords:** Chemotherapy, Neoangiogenesis, Immune tolerance, Antiangiogenesis, Antitumor immunity, Immunotherapy

## Abstract

Studies have shown that cancer requires two conditions for tumor progression: cancer cell proliferation and an environment permissive to and conditioned by malignancy. Chemotherapy aims to control the number and proliferation of cancer cells, but it does not effectively control the two best-known conditions of the tumor-permissive environment: neoangiogenesis and tolerogenic immunity. Many malignant diseases exhibit poor outcomes after treatment with chemotherapy. Therefore, we investigated the potential benefits of adding an induction regimen of antiangiogenesis and antitumor immunity to chemotherapy in poor outcome disease. In a prospective, randomized trial, we included patients with advanced, unresectable pancreatic adenocarcinomas, non-small cell lung cancer, or prostate cancer. Two groups of each primary condition were compared: group 1 (G1), *n* = 30, was treated with the standard chemotherapy and used as a control, and group 2 (G2), *n* = 30, was treated with chemotherapy plus an induction regimen of antiangiogenesis and antitumor immunity. This induction regimen included a low dose of metronomic cyclophosphamide, a high dose of Cox-2 inhibitor, granulocyte colony-stimulating factor, a sulfhydryl (SH) donor, and a hemoderivative that contained autologous tumor antigens released from patient tumors into the blood. After treatment, the G2 group demonstrated significantly longer survival, lower blood level of neoangiogenesis and immune-tolerance mediators, and higher blood levels of antiangiogenesis and antitumor immunity mediators compared with the G1 group. Toxicity and quality of life were not significantly different between the groups. In conclusion, in several advanced malignancies of different primary localizations, an increase in survival was observed by adding an induction regimen of antiangiogenesis and antitumor immunity to standard chemotherapy.

## Introduction

Classical chemotherapy, which exerts its antitumor activity by causing damage and inducing apoptosis in rapidly dividing cells, has been a corner stone in standard cancer treatment for several decades. The rationale for using classical chemotherapy is to kill malignant cells in order to reduce tumor size. However, this method has not provided satisfactory benefits for patients with advanced cancers and poor prognoses in terms of survival. Often, these patients experience disease progression after a short period of remission, if any, despite treatment with classical chemotherapy. This progression requires not only residual cancer cells, but also a biological response permissive to and conditioned by the malignancy, according to several reports in the current literature [1, 2]. In these reports, two broad components of the permissive biological response were identified: neoangiogenesis and tolerogenic immunity. Therefore, in addition to using treatments that kill cancer cells, targeting these additional components may also improve the antiprogressive efficacy of the treatments.

Recently, several clinical trials have attempted to control neoangiogenesis by incorporating antiangiogenic therapies into classical chemotherapy treatments for malignancies with poor prognoses. Improvements in progression-free survival have been shown in some cases. However, it is premature to draw conclusions about the overall survival benefits based on currently available evidence [3]. In order to optimize these results, it was suggested that agents that target both neoangiogenesis and tolerogenic immunity, and not neoangiogenesis alone, might provide a greater benefit as adjuvants of chemotherapy. Indeed, the relevance of the tolerogenic immune component in a permissive biological response of malignant progression has been highlighted in reports that identified tolerogenic immunity as an early, permanent, and common phenomenon of malignancies [4]. Researchers previously reported that some standard chemotherapies [5, 6], drugs used in non-cancer conditions [7, 8], and cancer vaccines [9] could switch angiogenesis and immune responses from a tumor progression/tolerance balance to an antiprogressive/antitumor balance when used at specific dosages within a particular regimen. Therefore, in this study, we tested the effect of combining a set of agents that have been reported to promote antiangiogenesis and switch tumor tolerogenic immunity to antitumor immunity with standard chemotherapy [2]. In order to determine the applicability of our approach in different cancers, we tested the regimen in three malignancies with recognized poor prognoses, high prevalence, and appropriate survival expectancy for this study, namely unresectable [10] locally advanced pancreatic cancer, non-small-cell lung cancer (NSCLC), and hormone-refractory metastatic prostate cancer.

Pancreatic cancer is a worldwide health problem, and surgery is currently the only potentially curative treatment. However, the number of newly diagnosed patients with surgically resectable pancreatic cancer is limited to 10–20 %. Locally advanced disease is observed in 15–20 % of patients, which is associated with a median survival time of 6–10 months. To date, chemotherapy only provides a marginal improvement in the overall survival for these patients. Similarly, lung cancer is the leading cause of cancer-related mortality for men and women worldwide. In the United States, 222,520 new cases of lung cancer were diagnosed in 2010, and 157,300 deaths resulted from the disease. Approximately 85 % of primary lung cancers are categorized as NSCLC, which includes the main histological subtypes of adenocarcinoma, squamous cell carcinoma, and large cell carcinoma. The majority of NSCLC patients present with advanced disease at diagnosis, and the survival rates are quite low. The overall survival for patients with unresectable NSCLC is generally 13–14 months after treatment. Lastly, prostate cancer is one of the most common solid tumors affecting men. It is the second most commonly diagnosed form of cancer and the sixth leading cause of cancer-related deaths among men worldwide. Once metastasized to distant organs, prostate cancer is incurable, leaving clinicians with palliative care as the only option for disease management. In their hormone-refractory stage, more than 84 % of prostate tumors metastasize, with a median patient survival of approximately 14 months. Therefore, in this study, we investigated the potential benefits of adding an induction regimen of antiangiogenesis and antitumor immunity to chemotherapy in poor outcome disease.

## Materials and methods

### Study design

A prospective, randomized, phase 1/2 trial was designed primarily to assess safety, tolerance, and preliminary efficacy of the combination of standard chemotherapy with the aforementioned, previously published treatment that switches both angiogenesis and immunity conditioning. This assessment was performed in patients with poor prognoses and unresectable malignancies of the pancreas, lung, or prostate. The study protocol was approved by the institutional review board and conducted in accordance with the Declaration of Helsinki [11]. Written informed consent was obtained from all patients at the time of enrollment.

For each primary localization, patients were included and randomly distributed in one of two groups: G1 (*n* = 30), which received standard chemotherapy for the cancer condition, and G2 (*n* = 30), which received standard chemotherapy and the antiangiogenesis and antitumor immunity induction regimen. The study design included a follow-up of 2 years. The primary endpoint was overall survival. Secondary endpoints were toxicity and quality of life.

All of the patients of the three primary localizations were regrouped into two cohorts: 90G1 (*n* = 90), which included patients who had only received standard chemotherapy for each primary localization, and 90G2 (*n* = 90), which included patients treated with the same chemotherapy and the induction regimen of antiangiogenesis and antitumor immunity agents. Blood concentrations of known mediators of angiogenesis and immunity were measured, and the series of values in the 90G1 and 90G2 cohorts were statistically compared.

### Patients

Inclusion criteria were as follows: patients with 18–65 years of age who were diagnosed with unresectable, histologically confirmed, pancreatic adenocarcinoma, NSCLC, or prostate cancer; who had a performance status 0–2 according to the Eastern Cooperative Oncology Group [12]; and who were expected to survive for at least 4 months. Organic functions required for inclusion were absolute neutrophil count ≥1,500/μL, lymphocyte count ≥1,000/μL, platelet count ≥100,000/μL, hemoglobin ≥8 g/dL, serum creatinine <1.5-fold of the upper limit of normal (ULN) value, alkaline phosphatase <3-fold, and bilirubin <1.5-fold of the ULN value. The included locally advanced pancreatic cancer patients had M0 metastatic status with locally advanced tumor and had undergone choledochoenteric bypass before inclusion. The included NSCLC patients had M0 metastatic status, locally advanced tumor, without epidermal growth factor receptor (EGFR) mutations. The hormone-refractory metastatic prostate cancer patients included were M1-stage disease. Exclusion criteria included patients who exhibited comorbidity requiring treatment, who were pregnant, and/or who could not complete the treatment regimen and follow-up.

### Treatments

#### Chemotherapy

The following standard chemotherapy treatments were used in this study: Gemcitabine (Gemzar), 1,000 mg/m^2^ I.V., on days 1, 8, 15 every 4 weeks for pancreatic cancer; Cisplatin (Paraplatin), 75 mg/m^2^ I.V., plus Docetaxel (Taxotere) 75 mg/m^2^ I.V., every 3 weeks for NSCLC; and docetaxel (Taxotere) 75 mg/m^2^ I.V., every 3 weeks for prostate cancer.

#### Induction regimen of antiangiogenesis and antitumor immunity

**Fig. 1:**
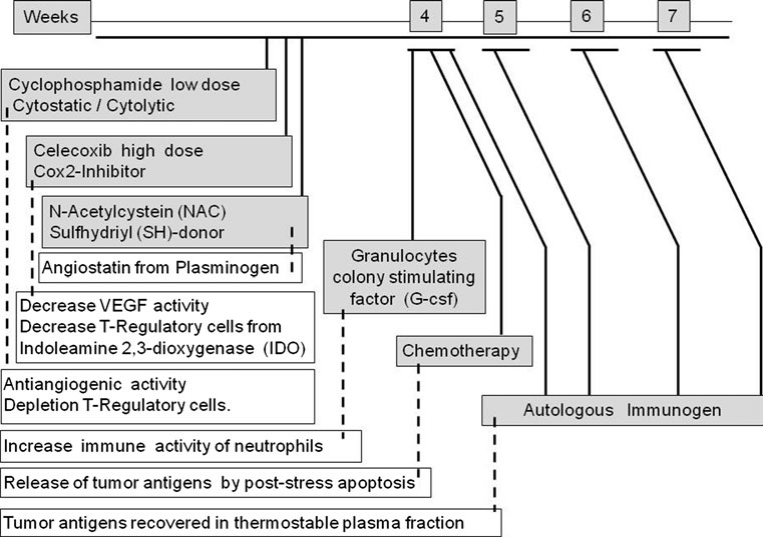
Induction regimen of antiangiogenic and antitumor immunity agents administered in an 8-week series. The *dashed lines* indicate the targeted mechanism proposed

In order to induce a switch of conditioning from the malignancy-induced neoangiogenesis and tolerogenic immunity to antiangiogenesis and antitumor immunity (Fig. 1), patients received oral cyclophosphamide (Cytoxan) 50 mg q.d., the Cox-2 inhibitor celecoxib (Celebrex) 400 mg b.i.d., and the sulfhydryl (SH) donor *N*-acetylcysteine (oral NAC) 400 mg b.i.d.

After the switch of conditioning, specific antitumor immunity was induced through subcutaneous immunization performed every 4 weeks using a thermostable autologous plasma fraction obtained from drawn blood. This fraction has been shown to contain tumor antigens released spontaneously and because of chemotherapy-induced apoptosis [13].

### Assessments

The following tests were performed on all patients prior to treatment (baseline) and 3 months after the start of treatment. The results were expressed as a percentage of baseline levels.

Delayed-type hypersensitivity (DTH) assay was performed by injecting an aliquot of the autologous hemoderivative used in the immunization to the volar surface of the forearms. An induration >5 mm after 48 h was considered a positive DTH response.

An IFN-ELISPot assay was used to assess for the presence of IFN-producing T-lymphocytes. Dendritic cells (DCs) were pulsed with autologous hemoderivative immunogen from patients and healthy donors as controls. Pulsed DCs were co-incubated with autologous T-cells for 40 h. The total number of T-cells per well was 5 × 10^4^. The number of IFN spots was measured automatically using ELISPot software (Carl Zeiss Vision). The frequency of tumor-reactive T-cells was calculated as follows: (number of spots in wells with immunogen-pulsed DCs − number of spots in control wells)/number of T-cells per well. Individuals were considered positive when the number of spots in the presence of DCs pulsed with immunogen was significantly higher than in control wells (*p* < 0.05).

Vascular endothelial growth factor (VEGF) and angiostatin (AT) levels in blood samples were determined by ELISA using standard laboratory techniques.

T-regulatory cells (T-Reg) were assessed by immunocytochemistry (IHC) and flow cytometry as CD4+CD25+ Foxp3+ by CD4+. Activated DCs (aDCs) were assessed by IHC as CD3+CD86+ by CD3+.

### Efficacy and safety

Survival was plotted in Kaplan–Meier curves, and the mean and standard deviation of time required to reach 50 % of survival were calculated. The difference between means in different treatment groups was analyzed using the log-rank test. Statistical significance was set at *p* = 0.05.

A safety evaluation included monitoring for hematological toxicity, nausea/vomiting, changes in liver function, changes in renal function, and CNS toxicity. Cardiac function was monitored by echocardiograms. Toxicities were graded according to Common Terminology Criteria for Adverse Events (CTCAE) v3.0 of the National Cancer Institute [14]. Quality of life was scored using the current core questionnaire of the EORTC QLQ-C30 [15].

## Results

**Fig. 2:**
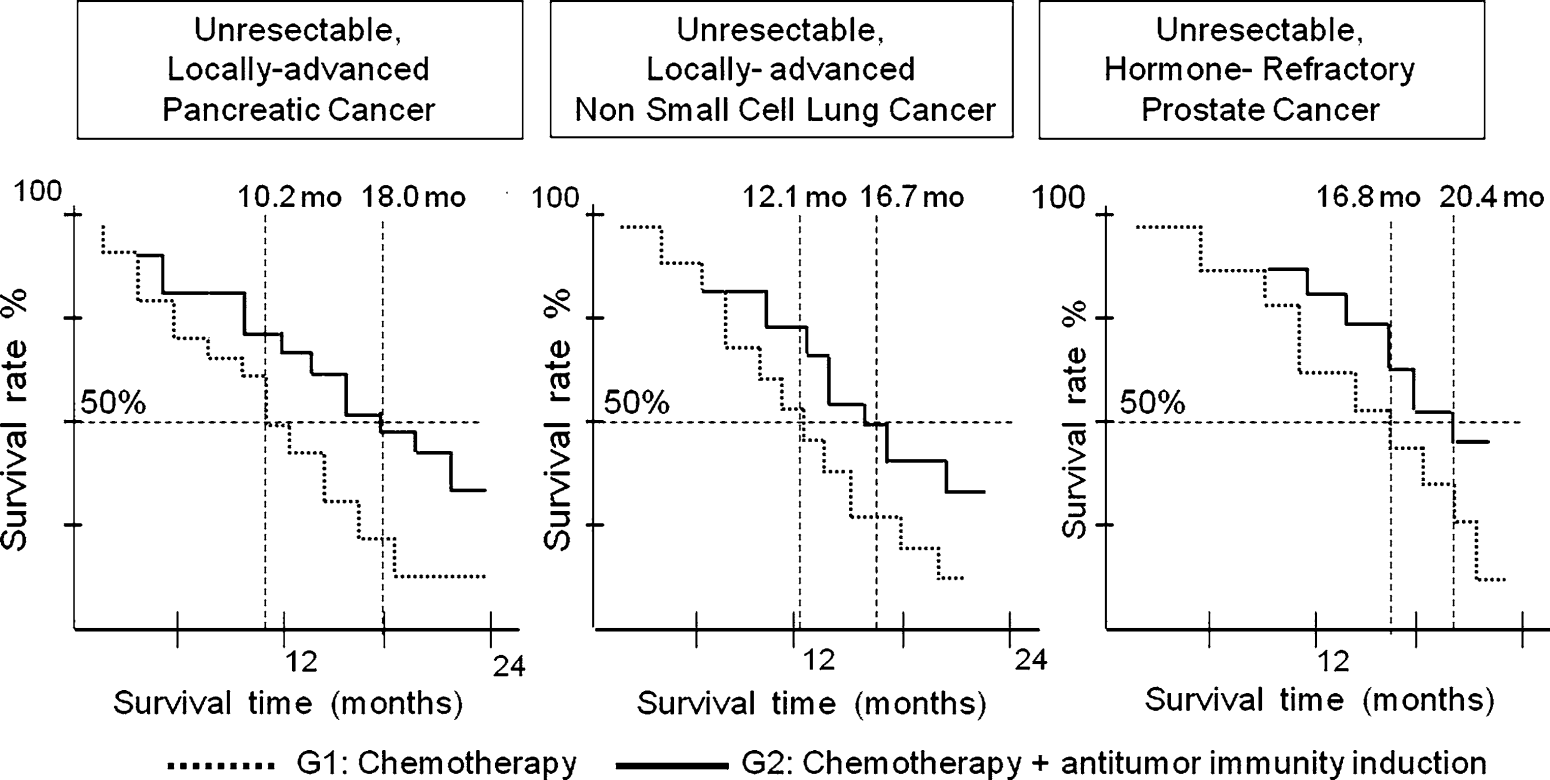
The Kaplan–Meier survival curves plotted for patients with pancreatic cancer, NSCLC, and prostate cancer with poor prognoses. *G1* groups of patients treated with standard chemotherapy; *G2* groups of patients treated with standard chemotherapy and an induction regimen of antiangiogenic and antitumor immunity agents. Mean survival is significantly longer for G2 than for G1 for patients with the three primary malignancies analyzed: 18.0 versus 10.2 months (log-rank, *p* = 0.036), 16.7 versus 12.1 months (log-rank, *p* = 0.042), and 20.4 versus 16.8 months (log-rank, *p* = 0.048) for pancreatic cancer, NSCLC, and prostate cancer, respectively

Figure 2 shows the Kaplan–Meier plot [16] estimates for survival of G1 and G2 patients for each cancer studied (pancreatic, NSCLC, and hormone-refractory prostate cancer). The addition of the tested regimen in G2, which is a recognized procedure for eliciting antiangiogenesis and antitumor immunity, improved the survival rate compared with G1 patients who were only treated with chemotherapy. The mean survival was significantly longer for G2 patients than for G1 patients for the three tumor types analyzed: 18.0 versus 10.2 months (log-rank, *p* = 0.036), 16.7 versus 12.1 months (log-rank, *p* = 0.042), and 20.4 versus 16.8 months (log-rank, *p* = 0.048) for pancreatic cancer, NSCLC, and prostate cancer, respectively.

**Fig. 3:**
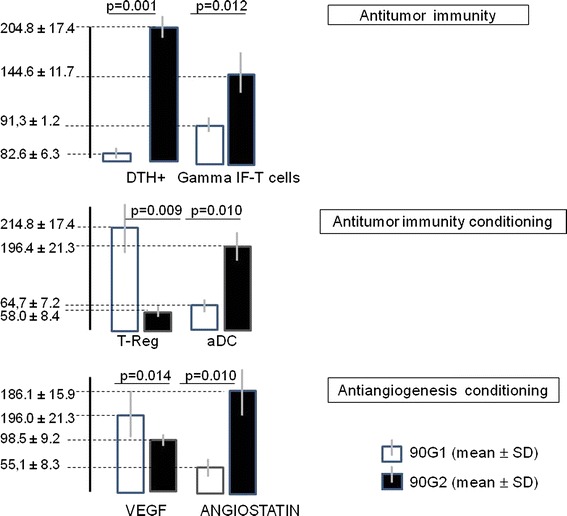
Percentage of baseline (pretreatment) values (mean ± SD) at 3 months of follow-up in the 90G1 cohort treated with standard chemotherapy compared with the 90G2 cohort treated with the same chemotherapy and an induction regimen of antiangiogenic and antitumor immunity agents. Antiangiogenesis was monitored by measuring VEGF and angiostatin blood levels. Antitumor immunity conditioning was determining by assessing the number and presence of T-Regs and aDCs. Antitumor immunity was tested with DTH and IFN-ELISPot assays challenged with an autologous hemoderivative containing tumor antigens

To interpret these findings, we also confirmed the efficacy of this regimen in the frame of this study for switching neoangiogenesis and tolerogenic immunity to antiangiogenesis and antitumor immunity. For this purpose, we assessed the percent change from baseline of markers of neoangiogenesis (VEGF), antiangiogenesis (AT), immunity response (aDC), and tolerogenic immunity (T-Reg) after 3 months of treatment. Figure 3 showed the results in the cohorts 90G1 and 90G2 expressed as a percentage of baseline levels. VEGF levels were significantly higher in 90G1 patients compared with 90G2 patients (196.0 ± 21.3 vs. 98.5 ± 9.2, respectively; *p* = 0.014). Moreover, AT reached levels significantly higher in patients in 90G2 compared with patients in 90G1 (186.1 ± 15.9 vs. 55.1 ± 8.3, respectively; *p* = 0.010). In addition, the T-Reg levels were significantly lower in 90G2 patients compared with 90G1 patients (58.0 ± 8.4 vs. 214.8 ± 17.4, respectively; *p* = 0.009). The levels of aDC increased in 90G2 patients compared with 90G1 patients (196.4 ± 21.3 vs. 64.7 ± 7.2, respectively; *p* = 0.010). Furthermore, we aimed to confirm that these changes in the immunity response conditioning and the immunization with the autologous hemoderivative were sufficient to allow for the emergence of antiautologous-tumor immunity. Using the autologous hemoderivative containing tumor antigens for the immune challenge, we performed a DTH test to assess for cellular-mediated immune responses and an IFN-ELISPot assay to measure for IFN-producing T-lymphocytes. We found that compared with baseline values, the percentage of positive DTH in 90G2 patients significantly increased after 3 months of therapy (204.8 ± 17.4), while the percentage significantly decreased slightly in patients of the 90G1 group (82.6 ± 6.3; *p* = 0.001). In addition, the percentage of the number of spots in the IFN-ELISPot assay was higher for the 90G2 patients compared with 90G1 patients after 3 months of therapy compared with baseline (144.6 ± 11.7 vs. 91.3 ± 1.2, respectively; *p* = 0.012).

**Fig. 4:**
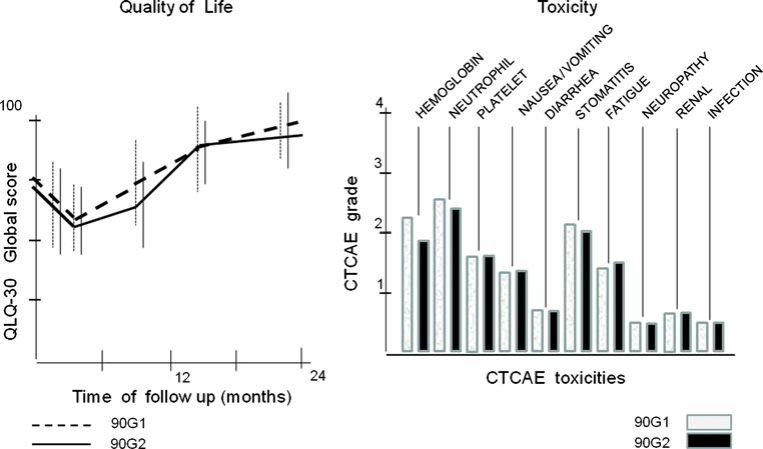
The safety and toxicity profiles of the 90G2 cohort treated with the induction regimen of antiangiogenic and antitumor immunity agents in combination with chemotherapy and the 90G1 cohort treated with standard chemotherapy alone were not statistically different in the 2-year follow-up period (*p* > 0.05). Toxicities were graded according to Common Terminology Criteria for Adverse Events (CTCAE) v3.0 of the National Cancer Institute. Quality of life was scored using the current core questionnaire of the EORTC QLQ-C30

As shown in Fig. 4, no significant differences (*p* > 0.05) were observed in toxicities or quality of life profiles between the two cohorts during the 2-year follow-up period. The toxicities and quality of life profiles observed in the cohort receiving the induction regimen of antiangiogenesis and antitumor immunity were as expected and related to the chemotherapy regimen.

## Discussion

It is now well accepted that carcinogenesis includes the conditioning of a patient’s biological response, including neo-angiogenesis and tolerogenic immunity, for disease progression to occur. This study aimed to explore the rationale of a complementary therapeutic approach that targets angiogenesis and immunity. In this trial, the analysis of two 30-patient groups of three different primary cancer types showed that survival was significantly improved when an induction regimen of antiangiogenesis and antitumor immunity was added to chemotherapy compared with chemotherapy alone. This survival improvement was observed in patients with advanced pancreatic cancer, NSCLC, and hormone-refractory prostate cancer. Interestingly, the degree of improvement, though varied, was significant for all three of the primary diseases assessed in this study, indicating that this approach has a general benefit and suggests that the pathogenic and therapeutic mechanisms involved are essential for malignancies.

The link between these effects on survival and the modulation of the biological response was shown by comparing a 90-patient cohort that was treated with only chemotherapy with a 90-patient cohort treated with chemotherapy plus the induction regimen of antiangiogenesis and antitumor immunity. Although separate clinical trials for each type of cancer would be beneficial for analysis purposes, we believe that this design was more effective for assessing the essential mechanism proposed for the development of malignancies. The comparability of the analyzed groups was possible due to enrollment of the same number of patients with the three tumor types in each group as well as the use of the same inclusion and exclusion criteria.

After 3 months of treatment, the assessment of angiogenesis and immunity mediators in blood showed a net increase in AT and VEGF levels as well as a net increase in aDC and T-Reg cells. These results are in agreement with a change in the conditioning of the biological response induced by malignancies, neo-angiogenesis, and tolerogenic immunity [17–19]. The conditioning becomes more antiangiogenic and less immune tolerogenic when chemotherapy is combined with an induction regimen of antiangiogenesis and antitumor immunity. This modulatory activity can be explained by the known properties of the agents included in this regimen [20]. Metronomic treatment with a low dose of cyclophosphamide has been shown to not only be antiangiogenic due to its antiproliferative activity upon endothelial cells, but also antitolerogenic by selectively depleting regulatory T-cells and restoring T and NK effector functions in immunity [21]. In addition, Cox-2 inhibitors interfere with VEGF expression in neoangiogenesis and also block indoleamine 2,3-dioxygenase (IDO) activity, which is required to generate the tolerogenic immunity of T-Regs [22]. Granulocyte colony-stimulating factor (G-CSF) increases the number of peripheral blood DCs and the expression of their activation markers [23], thereby improving the antigen processing and presentation (i.e., classical non-tolerogenic immunity). Furthermore, sulfhydryl (SH) donors improve the generation of angiostatin from autoproteolysis of plasmin [24], allowing tumor infiltration from the blood immune-competent cells [25]. Taken together, the properties of the drugs included in the induction regimen explain the tumor infiltration effects of the antiangiogenics and the non-tolerogenic immune-responder cell population. However, the generation of antitumor immunity requires not only immune-responder cells, but also a challenge of the immune system with tumor antigens. In the tested antiangiogenesis and antitumor immunity induction regimen, the tumor antigens were those released from tumors into the bloodstream by spontaneous [26–28] or chemotherapy-induced apoptosis [29, 30] of previously stressed cells [31]. Indeed, it has been reported that some of those tumor antigens released by tumors circulate as protected complexes with heat-shock proteins can induce vaccination against tumors [32–35] and can be recovered in a thermostable hemoderivative [13]. This hemoderivative was used as the tumor immunogen to challenge the conditioned immune-responder cells. The results of cell-mediated immune responses assessed by DTH and IFN-ELISPot assays indicated the efficiency of this immunogen to induce antitumor immunity. Introducing autologous antitumor immunity in cancer treatments, as previously stated [36], adds autologous tumor specificity, exposes the current tumor antigen library to the immune system, and provides immune memory. Taken together, the results of this study are compatible with the rationale of combining tumor cell killing with an induction regimen of antiangiogenesis and antitumor immunity.

## Conclusions

In advanced malignant diseases with poor prognoses that are treated with standard chemotherapy, the addition of an induction regimen of antiangiogenic and antitumor immunity agents that effectively switch the biological response from neoangiogenesis to antiangiogenesis and the immunity from permissive to antitumor immunity safely improved the survival of patients with three different tumor types in this study. Although these results are preliminary, they encourage further studies to confirm the clinical relevance of these findings.
